# Development and Field Evaluation of a Synthetic Mosquito Lure That Is More Attractive than Humans

**DOI:** 10.1371/journal.pone.0008951

**Published:** 2010-01-28

**Authors:** Fredros O. Okumu, Gerry F. Killeen, Sheila Ogoma, Lubandwa Biswaro, Renate C. Smallegange, Edgar Mbeyela, Emmanuel Titus, Cristina Munk, Hassan Ngonyani, Willem Takken, Hassan Mshinda, Wolfgang R. Mukabana, Sarah J. Moore

**Affiliations:** 1 Biomedical and Environmental Sciences Thematic Group, Ifakara Health Institute, Ifakara, Tanzania; 2 School of Biological Sciences, University of Nairobi, Nairobi, Kenya; 3 Disease Control and Vector Biology Unit, London School of Hygiene and Tropical Medicine, London, United Kingdom; 4 School of Biological Sciences, Durham University, Durham, United Kingdom; 5 Vector Group, Liverpool School of Tropical Medicine, Liverpool, United Kingdom; 6 College of Agriculture and Life Sciences, Cornell University, Ithaca, New York, United States of America; 7 Laboratory of Entomology, Wageningen University and Research Centre, Wageningen, The Netherlands; BMSI-A*STAR, Singapore

## Abstract

**Background:**

Disease transmitting mosquitoes locate humans and other blood hosts by identifying their characteristic odor profiles. Using their olfactory organs, the mosquitoes detect compounds present in human breath, sweat and skins, and use these as cues to locate and obtain blood from the humans. These odor compounds can be synthesized *in vitro*, then formulated to mimic humans. While some synthetic mosquito lures already exist, evidence supporting their utility is limited to laboratory settings, where long-range stimuli cannot be investigated.

**Methodology and Principal Findings:**

Here we report the development and field evaluation of an odor blend consisting of known mosquito attractants namely carbon dioxide, ammonia and carboxylic acids, which was optimized at distances comparable with attractive ranges of humans to mosquitoes. Binary choice assays were conducted inside a large-cage semi-field enclosure using attractant-baited traps placed 20 m apart. This enabled high-throughput optimization of concentrations at which the individual candidate attractants needed to be added so as to obtain a blend maximally attractive to laboratory-reared *An. gambiae*. To determine whether wild mosquitoes would also be attracted to this synthetic odor blend and to compare it with whole humans under epidemiologically relevant conditions, field experiments were conducted inside experimental huts, where the blend was compared with 10 different adult male volunteers (20-34 years old). The blend attracted 3 to 5 times more mosquitoes than humans when the two baits were in different experimental huts (10–100 metres apart), but was equally or less attractive than humans when compared side by side within same huts.

**Conclusion and Significance:**

This highly attractive substitute for human baits might enable development of technologies for trapping mosquitoes in numbers sufficient to prevent rather than merely monitor transmission of mosquito-borne diseases.

## Introduction

Mosquitoes rely on biochemical cues to find essential resources such as human hosts, mates and suitable sites to lay their eggs [1]. African mosquitoes such as *Anopheles gambiae sensu stricto* and *Anopheles funestus* are efficient vectors of human malaria because they are highly adapted to preferentially feed on humans [2]. Using their highly sensitive olfactory organs, these mosquitoes can select more attractive persons over less attractive ones by identifying chemicals present in breath, sweat and other skin emanations originating from the persons [3], [4]. Though not adequately understood, these evolutionary host preferences may benefit the mosquitoes in a number of ways including the identification of hosts with more nutritive blood, or those who are less defensive against mosquito bites [3], [4]. Examples of these attractive chemicals include carbon dioxide (CO_2_), the ultimate catabolite of vertebrate respiratory processes, L-lactic acid, which is produced through anaerobic glycolysis in human eccrine glands, and ammonia, also a component of human sweat [5], [6], [7]. Human skin also secretes triglycerides, which can be broken down to several behaviorally active fatty acids by skin surface microflora [3].

Many of these odorants can be synthesized *in vitro* and therefore they can be reformulated to produce mixtures that mimic real humans to lure mosquitoes [8], [9]. While some synthetic odor mixtures already exist which can lure host-seeking mosquitoes, evidence supporting their utility is largely limited to short range evaluations inside laboratories, where the crucial role of long-range stimuli is artificially negated [10], [11], [12]. Other recent successes include the formulation of synthetic odor blends which can match the attractiveness of humans for field populations of certain mosquitoes transmitting malaria [13], dengue and yellow fever [14], but none of these improves upon humans for priority vector species. The former example [13] does surpass humans for zoophagic species which transmit several neglected tropical diseases but this inevitably biases the sample of mosquitoes obtained so that they cannot be considered epidemiologically representative for surveillance of human exposure to the full range of mosquito-borne pathogens.

**Figure 1:**
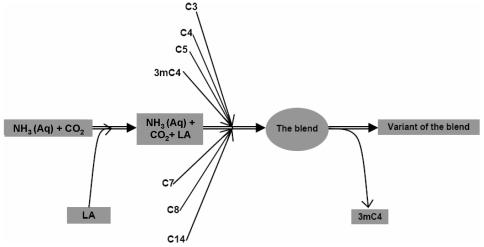
A conceptual model summarizing the development of the odor blend. The process began with a weakly attractive mixture containing 2.5% aqueous ammonia and CO_2_ gas flowing at 500 ml/min, which was enhanced by adding 85% L-lactic acid (LA). Onto the resulting mixture, each of the other aliphatic carboxylic acids was added separately, each of them at their optimally attractive concentrations. The blend therefore consisted of the CO_2_ gas plus hydrous solutions of ammonia (2.5%) and L-lactic acid (85%), and the other aliphatic carboxylic acids at their respective optimum concentrations as follows: propionic acid (C3) at 0.1%, butanoic acid (C4) at 1%, pentanoic acid (C5) at 0.01%, 3-methylbutanoic acid (3mC4) at 0.001%, heptanoic acid (C7) at 0.01%, octanoic acid (C8) at 0.01% and tetradecanoic acid (C14) at 0.01%. Finally, a variant of the blend was formulated by removing 3mC4.

Here we report on the development and field evaluation of a highly attractive odor blend consisting of synthetic versions of known mosquito attractants [6], [12], [13], [15]: carbon dioxide, ammonia, L-lactic acid and seven other aliphatic carboxylic acids, namely propionic acid, butanoic acid, pentanoic acid, 3-methylbutanoic acid, heptanoic acid, octanoic acid and tetradecanoic acid. The conceptual process followed in developing the blend is illustrated in Fig. 1.

## Materials and Methods

### Mosquitoes

The semi-field assays were conducted using laboratory-reared *An. gambiae s.s.*, which had originally been obtained from Njagi village in Kilombero district, southern Tanzania. This colony had originally been established in 1997, but was recently restocked in 2008. The mosquito larvae were fed on Tetramin® fish food and maintained at a temperature of 27±1°C. The adult mosquitoes were kept inside a separate room, where temperatures were maintained at 27°C and relative humidity at 70–90% and were fed on 10% glucose solution. To sustain the colony, the adult female mosquitoes were fed also on human blood (by way of adult volunteers regularly inserting their arms inside the mosquito cages for 5–10 minutes every two days). The insectary was set to a photoperiod of 12 hours darkness and 12 hours light.

### The Semi-Field System

All behavioral assays aimed at the formulation and optimization of the synthetic odor blend were conducted within a semi-field enclosure (screen house), at the Ifakara Health Institute [16]. The screen house has three 200 m^2^ compartments, one of which was used for this research.

### Formulation and Optimization of the Synthetic Odor Blend

The blend was developed through a sequential procedure (Fig. 1), starting with a weakly attractive primary mixture consisting of 2.5% ammonia solution and 500 ml/min of CO_2_ gas. Onto this primary mixture, L-lactic acid was first added followed by the other aliphatic carboxylic acids initially one at a time to determine their optimal concentrations, and then jointly at their optimal concentrations to create the final blend. Whenever each of the candidate compounds were added, we iteratively varied its concentrations until the point when the resulting mixture was maximally competitive, in terms of its attractiveness to laboratory reared mosquitoes relative to the original mixture. This final concentration was considered the optimum for each respective candidate compound.

L-Lactic acid was the first to be added onto the primary mixture. The treatment trap was baited with the primary mixture plus different concentrations of L-lactic acid. The control trap on the other hand was baited with only the primary mixture. For each concentration of L-lactic acid, four replicates were conducted each lasting six hours, and between which we rotated the positions of the traps so as to minimize directional bias. The most significant improvement in attractiveness was determined to occur when L-lactic acid was added at 85% concentration (which was the undiluted formulation of L-lactic acid as purchased from the manufacturer).

Previously, the synergistic effect of L-lactic acid when combined with ammonia and a blend of carboxylic acids, has been demonstrated [17], [18]. Therefore in the rest of our assays, all other aliphatic carboxylic acids were all tested in combination with 85% L-lactic acid, each time comparing the resulting mixture with the original mixture as before. In the case of these other carboxylic acids, the treatment trap was therefore baited with: 1) the primary mixture, 2) undiluted L-lactic acid and 3) an iteratively selected concentration of a selected carboxylic acid, while the control trap was baited with only the primary mixture. For each carboxylic acid, the optimal concentration was determined by iterating until the point when the resulting mixture was maximally attractive relative to the primary mixture.

After the optimal concentrations for all the carboxylic acids had been determined, all the compounds were added to the primary mixture at those respective concentrations to form the final synthetic odor blend. The synthetic blend therefore consisted of CO_2_ gas flowing at 500 ml/min plus hydrous solutions of ammonia (2.5%), L-lactic acid (85%), and the other aliphatic carboxylic acids: propionic acid (C3) at 0.1%, butanoic acid (C4) at 1%, pentanoic acid (C5) at 0.01%, 3-methylbutanoic acid (3mC4) at 0.001%, heptanoic acid (C7) at 0.01%, octanoic acid (C8) at 0.01% and tetradecanoic acid (C14) at 0.01%. Finally, a variant of the blend was formulated by removing the unpleasant smelling 3mC4 in an attempt to improve the appeal of the blend to potential users.

### Semi-Field Experiment Conducted to Compare the Attractiveness of the Synthetic Odor Blend with the Attractiveness of Natural Host Odors

The blend was first tested against a combination of CO_2_ gas and natural human foot odors collected in worn nylon socks, a technique previously used to trap *An. gambiae s.s* in a similar semi-field system [19]. To collect the foot odors, the nylon socks were worn for 10 hours by a 26 year old male volunteer prior to the experiment. Binary tests were conducted in which one trap was baited with the synthetic blend and the other trap baited with a worn nylon sock and 500 ml/min of CO_2_. Fourteen replicates, each lasting six hours, were conducted, swapping the positions of the two traps between replicates. The proportion of all responding mosquitoes that were caught in either trap was used to determine the attractiveness of the blend relative to the attractiveness of worn nylon sock plus CO_2_ (File S1).

### Field Experiments Conducted to Evaluate the Blend against Wild Mosquitoes

#### Study village

The field study was conducted in Lupiro Village (8.01°S and 36.63°E), Ulanga District, in the south eastern part of Tanzania. The village lies 300 metres above sea level, and experiences very high malaria transmission, recently reported to average approximately 400 infectious bites per year [20].

#### Ethics statement

After a full explanation of the risks involved and the objectives of the study, written informed consent was obtained from the volunteers, all of whom were male and aged between 20 to 34 years old. When inside the huts during the experimental nights, all volunteers slept under bed nets and were guaranteed immediate access to treatment including weekly screening for malaria parasites by light microscopy and treatment with artemether-lumefantrine; however no participant was affected during the study. Ethical approval for the study was obtained from Medical Research Coordination Committee of the National Institute for Medical Research of the United Republic of Tanzania (NIMR/HQ/R.8a/Vol.IX/710).

#### Comparing the number of mosquitoes that enter huts baited with the synthetic blend versus number of mosquitoes that enter huts in which there are human volunteers

To compare the synthetic odor blend with whole humans under epidemiologically relevant conditions, and determine whether wild mosquitoes would also be attracted to this it, field experiments were conducted in which the blend was compared with 10 different adult male volunteers (20–34 years old). We used 4 specially-designed experimental huts (Fig. S4) located 10 to 100 metres apart in a malaria-endemic village in rural Tanzania. The experimental huts were located at the edge of the village, so that they were between the main breeding grounds (a perennial rice field) and the human houses, and also so that they were at least 50 metres away from the nearest human house. Each night, 2 human volunteers slept under non-insecticidal bed nets inside 2 separate huts. In the third hut, the synthetic odor blend was dispensed by evaporation in a continuous air plume, also from under a non-insecticidal bed net. The fourth hut had a variant of this blend which lacked one constituent, 3-methyl-butanoic acid (3mC4). This constituent was the most unpleasant smelling, so it was expected that its exclusion would improve the acceptability of the odor blend. The blend and its variant were dispensed using nylon strips inserted in the attractant plume tube of an MM-X® trap (a counter flow geometry trap made by American Biophysics Corporation) and CO_2_ gas was added to the trap using rubber tubing (Figs. S1-S3). This odor dispensing method has been described in detail in Okumu *et al* [21].

In these field tests, the MM-X® was used only to dispense the blend but not to trap mosquitoes; its collection tube was closed and the exhausted fan disabled so as to eliminate suction. Instead, a different trap, the Centres for Disease Control Light Trap (CDC-LT), was set up in all the experimental huts to catch mosquitoes that enter. The light traps were placed beside the bed nets (from under which the odor cues from human or blend were emanating), such that it was one metre from the floor. To minimize any positional bias, the set up was rotated nightly so that at the end of a four-day rotation, the blend, its variant, and each volunteer had been to every hut. Also by the end of each rotation, the blend and its variant had been tested against humans four different times. Then a new pair of volunteers was recruited and the test repeated for another four nights. This way, the blend and its variant were each tested against 10 different humans (5 pairs) over 20 different nights. All experiments were conducted between 7.00pm to 7.00am, during which time mosquitoes entering the huts were trapped; and each morning, the mosquitoes were sorted into different taxa and counted.

#### Comparing the number of mosquitoes attracted to the synthetic odor blend versus the number of mosquitoes attracted to humans when both the synthetic blend and the human are located inside the same hut

We introduced the blend into two experimental huts in which there were single human volunteers sleeping under non-insecticidal bed-nets, such that in each hut, the blend and the human volunteer were positioned four meters apart. Two light traps were set up to collect mosquitoes inside each hut: one of the light traps near the human volunteer, and the other near the blend. In another two experimental huts, we set up a similar arrangement with single human volunteers but without introducing the blend. Instead, a blank unbaited MM-X® was set up at a similar location as in the first two huts, and similarly two light traps were set up in these huts. The volunteers remained in the same huts but the synthetic blend was rotated between the huts every night so as to account for any positional bias on mosquito catches. Again, for each experimental night mosquitoes entering the huts were trapped between 7.00pm and 7.00am. The mosquitoes were sorted into different taxa and counted daily. These experiments were repeated for a total of sixteen nights.

### Molecular Analysis

A total of 600 *An. gambiae sensu lato* mosquitoes were selected for further identification using ribosomal DNA-polymerase chain reaction [22].To constitute this total sample, 200 females were selected at random from catches obtained with each bait type.

### Data Analysis

Data from the semi-field experiments were analyzed by logistic regression using SPSS version 15 (SPSS Inc, Chicago). With regards to the data from the field experiments, we compared the arithmetic means of all the mosquito catches per hut per night and illustrated these using simple bar graphs with error bars showing 95% confidence intervals. Further analysis was conducted using General Linear Models (GLM) as follows as follows: Mosquito catches were modelled as a function of two fixed factors, bait and hut, treating day as a random variable to reflect daily fluctuations in mosquito numbers. Also, due to the heterogeneity of the mosquito counts, the data was log transformed to make it amenable to assumptions of the standard normal distribution.

## Results

High-throughput determination of optimal concentrations of individual candidate attractants was successfully achieved through the binary choice assays in the semi-field enclosure. These were the concentrations at which the individual candidate attractants needed to be added so as to obtain a blend maximally attractive to laboratory-reared *An. gambiae s.s.* When the resulting blend was tested within the semi-field enclosure against a combination of natural human foot odors collected in worn nylon socks and CO_2_ gas, it caught 46.5% (38.0%–55.9%) of all responding mosquitoes, indicating no difference between the two odor sources (P = 0.408).

**Figure 2:**
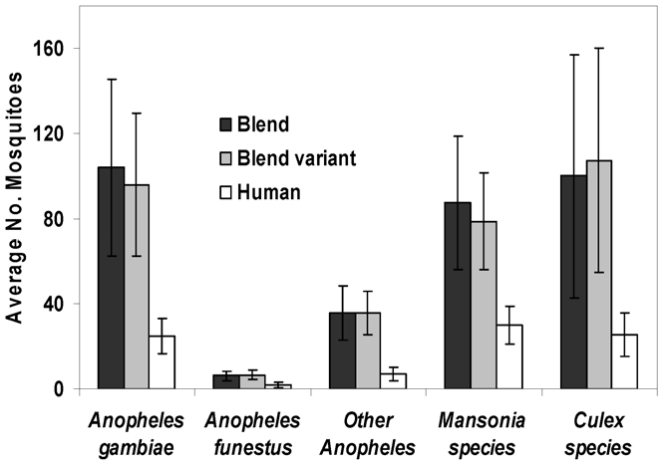
Long range performance of the synthetic blend and its variant. Average number of mosquitoes caught per night, inside experimental huts whenever the blend, its variant (without 3-methyl butanoic acid) or a human volunteer was inside the hut. There were significantly more mosquitoes caught in huts baited with the blend or its variant than in huts baited with humans (P<0.001) as determined by General Linear Model using SPSS version 15 (SPSS Inc.). However, no difference was observed between the blend and its variant (P>0.05). The error bars represent 95% confidence intervals.

In the field experiments conducted to compare our synthetic blend with humans, more mosquitoes were trapped inside the huts with either variant of the synthetic blend than inside the huts in which human volunteers slept (F = 66.025, P<0.001). With or without 3mC4, the blend attracted approximately 3.9 times more *An. gambiae sensu lato* (morphologically indistinguishable members of the species complex of which *An. gambiae* is a member), 3.1 times more *An. funestus*, 5.2 times more of other *Anopheles* species, 4.2 times more *Culex* mosquitoes and 2.7 times more *Mansonia* mosquitoes than an average human volunteer (Fig. 2).

**Figure 3:**
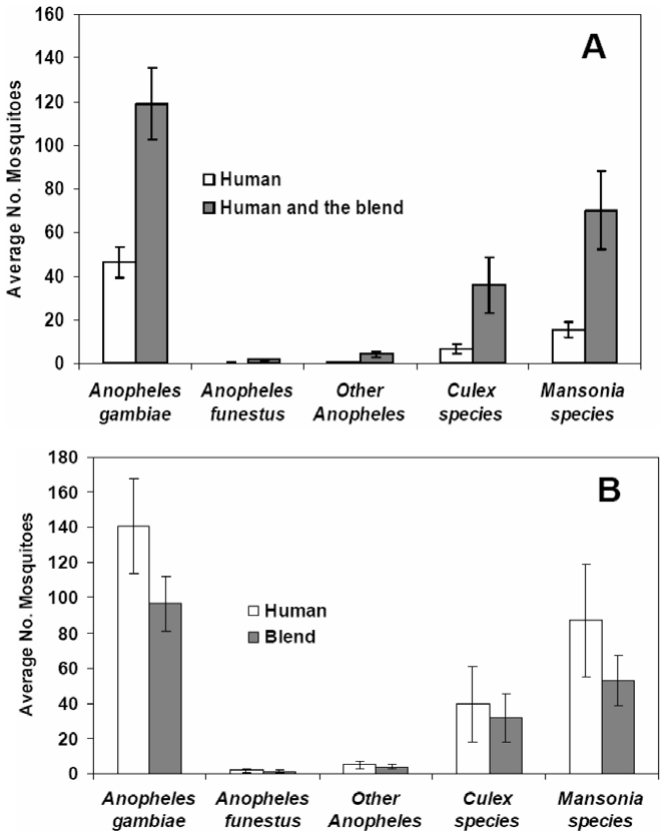
Short range performance of the synthetic blend. Addition of the synthetic blend into huts occupied by human volunteers significantly increased the number of mosquitoes caught in the huts compared to huts with only a human volunteer (A). However, once the mosquitoes were inside the huts, preferences for either bait type were similar for *An. funestus* (P = 0.179) and the other unidentified *Anopheles* mosquitoes (P = 0.82) but humans remained significantly more attractive to *An. gambiae s.l.* (P<0.001), *Culex* (P = 0.045) and *Mansonia* mosquitoes (P<0.001) (B), by General Linear Model using SPSS version 15 (SPSS Inc.). The error bars represent 95% confidence intervals.

In the other separate experiment, where the full blend was placed inside the same huts occupied by human volunteers, the number of mosquitoes of any species entering those huts was increased compared to huts in which there were only human volunteers. There were 2.6 times more *An. gambiae s.l*, 5.5 times more *Culex* and 4.6 times more *Mansonia* mosquitoes (Fig. 3A). However, once the mosquitoes had entered the huts, significantly more *An. gambiae s.l.* (P<0.001), *Culex* (P = 0.045) and *Mansonia* mosquitoes (P<0.001) were attracted to the humans than to the synthetic blend and similar but non-significant trends were observed for *An. funestus* (P = 0.179) and the assortment of other *Anopheles* mosquitoes (P = 0.82) present in the study village (Fig. 3B).

Molecular identification of *An. gambiae s.l.* mosquitoes caught in the experiment described in Fig. 1 showed that 97.8% (405/414) were *An. arabiensis*, with the remainder being *An. gambiae s.s.*, and this proportion did not vary with bait type (F = 2.96, P = 0.53). Furthermore, unlike previous blends tested in Africa [13], the proportions of mosquitoes caught which were accounted for by the various mosquito taxa were essentially identical to those obtained with human baits (χ^2^ = 2.94, P = 0.938), so these synthetic lures appear to representatively sample a wide range of human-biting mosquitoes with high sensitivity (Table 1).

**Table 1:** Proportions of various mosquito taxa making up the total mosquito catches in experimental huts baited with the blend, its variant (without 3-methylbutanoic acid) or humans.

	*Anopheles gambiae*	*Anopheles funestus*	Other *Anopheles*	*Mansonia* species	*Culex* species	Totals
Blend	31% (2082)	2% (125)	11% (715)	26% (1998)	30% (1748)	100% (6668)
Blend variant	30% (1921)	2% (132)	11% (712)	24% (2151)	33% (1576)	100% (6492)
Human	28% (991)	2% (78)	8% (281)	34% (1015)	28% (1200)	100% (3565)

Numbers in parentheses refer to the total number of mosquitoes caught during the entire field study.

## Discussion

This study greatly improves upon some of the recent successes with odor based technologies [10], [13], [14] and therefore represents a significant advancement in attempts to develop synthetic lures, which would effectively compete against humans for host seeking mosquitoes in actual field settings. Similar to previous field evaluations of synthetic blends for attracting *Aedes aegypti*, the principle vector of yellow fever and dengue [10], [14], [23], and *Anopheles gambiae*, the most important vector of malaria globally [13], our findings confirm that it is possible to formulate synthetic odor blends which attract as many mosquitoes as human odors, even without including all the biologically active components naturally found in the human emanations. In this study, we did not assume that a ‘surrogate human’ would consist of only the few compounds that we included. Instead, we expect that it would be possible to improve upon this simple mixture of carboxylic acids, ammonia and carbon dioxide by adding other attractants from different chemical groups, for example ketones and aldehydes, as well as physical cues such as heat and color.

Also, it appears that similar to results of previous studies [10], [24], preferences of mosquitoes towards any one of two different stimuli are dependent upon distances between those stimuli, i.e. whether the stimuli are in direct short range competition or whether they are far apart and have completely separated odor plumes (so that they are not perceived by the host seeking mosquitoes as two competing odour sources). Whereas huts baited with the synthetic blend or its variant had more mosquitoes than those huts with humans, many of the vectors when presented with the two odor sources side by side within the same hut appeared to retain their preferences for humans. On the basis of this observation and also because the experimental huts were located 10–100 metres apart, this synthetic blend may best be described as a medium to long range attractant rather than as a short range attractant [24].

The particular mosquito species caught in this study included three primary malaria vectors (*An. gambiae sensu stricto*, *An. arabiensis* and *An. funestus*) as well as carriers of Bancroftian filariasis and several arboviruses (*Culex pipiens quinquefasciatus*, *Cx. univattus*, *Cx. theileri*, *Cx. Rubinotus and Mansonia africana*), all of which are common and relevant across Africa (Ogoma *et al*., Unpublished). Therefore, although the synthetic blend was originally optimized using only laboratory-reared *An. gambiae sensu stricto*, it can be considered a general attractant that is equally effective for use against a range of other important human-biting mosquito species in the field.

This highly attractive and broad spectrum odor blend has many potential applications because it can substitute for human subjects as baits in mosquito traps, or as decoy hosts to lure away mosquitoes which would otherwise bite and possibly transmit pathogens to humans. The availability of such a consistent and effective bait for representative sampling a wide range of host-seeking mosquitoes means that it should be possible to measure human exposure to mosquito-borne infections without the need for human volunteers. The necessity to use human volunteers as baits in mosquito traps inevitably limits their compactness, consistency, cost and safety, and has restricted the development of sustainable mosquito surveillance systems, especially in resource-limited countries [25].

The attractants might also be applied for mass trapping of mosquitoes so as to reduce, rather than merely monitor, transmission of the various pathogens that the mosquitoes carry. Such mass trapping operations would simultaneously suppress nuisance bites from abundant Culicine mosquito species, thereby enhancing community uptake of such technologies. Another potential application of this odor blend is to combine them with existing malaria control tools using push-pull strategies similar to those commonly practiced in agricultural pest management [26], [27]. For example, where people use insecticide treated nets (ITNs) or indoor residual spraying with insecticides (IRS) which apart from being lethal targets also deter mosquitoes from entering houses, odor baited traps could be strategically located so as to trap the mosquitoes deterred from dwellings. Similarly, this odor blend could be used to lure host-seeking vectors to lethal outdoor targets such as surfaces treated with insecticides, a strategy that has been successfully applied to control tsetse flies [28].

One priority area for application of this or similar lures would be for malaria control, where there is currently an over-reliance on insecticide-based and intra-domiciliary methods, specifically ITNs and IRS. Mathematical simulations using adaptations of established transmission models [29], suggest that appropriate trap devices baited with these lures could reduce exposure to malaria, and effectively complement ITNs in areas of highly intense malaria transmission in Africa (Okumu *et al.*, Unpublished).

The fact that this synthetic blend greatly increased mosquito densities inside huts, yet humans sleeping in those huts remained equally or more attractive to the mosquitoes, necessitates careful consideration of safety and ethics whenever this or similar long-range attractants are used. Trap devices containing these blends should not be positioned in or near human habitations because this can increase exposure to mosquito bites and mosquito-borne pathogens. Our observations, combined with measurements of the attractive range of mammalian hosts to mosquitoes [30], imply that such devices must be located at least 30 meters away from human houses, a concept which can actually be exploited by mosquito control programs to target specific areas where mosquitoes are most abundant, notably the periphery of human settlements, thus enhancing impacts of such programs [31], [32]. The observation that the blend acts at medium-to-long range also suggests that optimization of behaviorally active formulations in even larger semi-field systems, followed by both short-range and long-range choice tests in the field, could accelerate and enhance the development of even more potent synthetic lures.

### Conclusion

We conclude that this synthetic odor blend has potential to be used for reproducible and exposure-free sampling of human-biting mosquitoes and also might be developed into high-impact, environmentally-friendly interventions against mosquitoes and diseases that they transmit. Added advantages of such technologies would include possibilities to target mosquitoes that bite outdoors or early in the evening before people go to bed and even diurnal vectors such as *Aedes aegypti* that often attack their victims far away from their houses [33]. Finally, because of the intimate dependence of mosquitoes upon their specialized host-seeking behaviors to obtain blood and survive, it is unlikely that odor-based technologies would be affected by physiological resistance among mosquitoes, as is common with insecticide based methods [34].

## Supporting Information

File S1 Supporting file for materials and methods section. This file contains additional information on candidate odor compounds, the trapping devices used, description of odor dispensing methods and statistical analysis used.
(0.03 MB DOC)

Figure S1 Picture (A) and Drawing (B) of the MM-X® trap. © American Biophysics Corporation.
(1.08 MB TIF)

Figure S2 Installation of the MM-X® trap showing how CO2, one of the blend constituents, was delivered to the trap.
(4.53 MB TIF)

Figure S3 Nylon strips to dispensing the synthetic odor blend. The strips were soaked in the individual test compounds, then removed and kept at 24°C for 4 to 6 hours, so that they were semi-dry by the start of the experiment. To bait the traps, the strips were batched together and inserted into the attractant plume tube of the MM-X® trap.
(2.10 MB TIF)

Figure S4 Experimental huts used for field evaluation of the synthetic blend. Four of these huts were used. The experimental huts were similar in average-dimension and shape to the local huts in the study area. They had a galvanized iron frame-work and corrugated iron sheet roofs overlaid with thatch walls were made of canvas on the outside and wood panels coated with mud on the inside. Each hut had one door, two windows and open eaves all round, similar to the local huts, and the daily indoor temperatures in the experimental huts were comparable to that in the local huts, averaging at 28°C. The four huts used for this study were positioned pair wise, such that the distance between two huts of the same pair was 10metres while the distance between the pairs was approximately 100metres.
(6.94 MB TIF)
