# Supplementary material for: Development and Field Evaluation of a Synthetic Mosquito Lure That Is More Attractive than Humans
Source: PLoS One. 2010 Jan 28;5(1):e8951. doi: 10.1371/journal.pone.0008951 (PMC2812511; doi:10.1371/journal.pone.0008951)
Supplement: File S1 — Supporting file for materials and methods section. This file contains additional information on candidate odor compounds, the trapping devices used, description of odor dispensing methods and statistical analysis used. (0.03 MB DOC) [file pone.0008951.s001.doc]

**Okumu et al: Supplementary Online Material**

**SUPPLEMENTARY METHODS**

**Candidate odor compounds:** The test compounds were all synthetically produced and included carbon dioxide (CO_2_) gas, aqueous ammonia (NH_3_OH), L-lactic acid, propionic acid (C3), butanoic acid (C4), pentanoic acid (C5), 3-methyl-butanoic acid (3mC4), heptanoic acid (C7), octanoic acid (C8) and tetradecanoic acid (C14). Ammonia was available as 25% aqueous solution while L-Lactic acid was available as 85% solution also aqueous. The other carboxylic acids were available as absolute formulations with purity levels ranging between 99.7% and 99.9%. The CO_2_ gas was available only as industrial grade and therefore its exact purity was difficult to ascertain. All the compounds were purchased from Sigma Aldrich® (United Kingdom), except CO_2_ which was supplied by Tanzania Oxygen Company Ltd®. In these studies CO_2_ was added to uniformly activate the mosquitoes but also as a synergist to improve the attractiveness of the other compounds (34). Distilled water was incorporated both as a solvent for ammonia, L-lactic acid and the aliphatic carboxylic acids C3–C7, but also to raise humidity levels in the odor plumes. The other carboxylic acids C8 and C14 were dissolved in ethanol.

**Trapping device used in the semi-field experiments**: We used a counter flow geometry trap (the MM-X® model) made by the American Biophysics Corporation. This trap (Fig. S1), consists of an oval shaped plastic casing (the collection container) enclosing an extended inner tubing where the bait is inserted (the attractant plume tube). It has two fans blowing air in opposite directions. The smaller fan (the attractant plume fan) located directly on top of the attractant plume tube blows air out while the larger fan (the exhaust fan), which is located near the top of the trap sucks air upwards through the trap, thereby creating a counter current suction mechanism. Attracted mosquitoes trace the path of the expelled air current, which carries the volatiles from the bait. When the insects reach near the lower end of the trap, they are sucked into the collection container by the more powerful current of the exhaust fan. At the end of the experiment, the collection tube is closed using a plastic seal after which the trap is disconnected from the 12 volt battery that powers it. To evaluate any two different synthetic odor blends, the MMX traps baited with the distinct odorants were placed in the opposite sides of the screen house so that they are 20 metres apart (Fig. S2).

**Method of dispensing the odorants during the field experiments:** The blend was delivered with its constituents separated but in such a way that the volatiles from these constituents were adequately mixed in the air plume coming out of the MM-X® trap (Figs. S1 and S3). Here, the MM-X® trap was used merely to dispense but not to trap any mosquitoes. The collection contained was always covered and the trap itself under a net, during the field experiments to ensure no mosquito enters them.

To dispense the blend, we used a method consisting of nylon strips as follows: nylon fabric made of 15 denier microfibres (90% polyamide and 10% spandex) and having a total surface area equal to that of a worn sock, was cut into 9 equal sized strips (Fig. S3). The CO_2_ gas was however delivered directly to the MM-X® using rubber tubing (Fig. S2).

**Additional information on statistical analysis:** Optimally attractive concentrations of the candidate compounds were determined and quantified as a relative improvement on the attractiveness of the control. The preference by mosquitoes to fly to the treatment trap or to the control trap was coded as 1 and 0 respectively, and then weighted by the number of mosquitoes caught per trap. Data was fitted to a binary logistic regression and the probability of mosquitoes being caught in treatment trap (P_t_) was estimated as a function of the categorical variables, trap location (side of the screen house) and phase of the night (whether the replicate had been conducted in the first 6 hours of the night or in the second six hours of the night). The intercept of the logistic regression equation was exponentiated to determine the odds of mosquitoes going to the treatment trap as opposed to the control trap. Finally, the probability that mosquitoes prefer odors from test blend was calculated as: Probability = odds/(1+odds).

For the field experiments, we compared the arithmetic means of all the mosquito catches per hut per night and illustrated these using simple bar graphs with error bars showing 95% confidence intervals. To determine the extent to which either bait was more attractive than the other, the field data was fitted to Generalized Linear Models as follows: Mosquito catches were modeled as a function of two fixed factors, bait and hut, treating day as a random variable to reflect daily fluctuations in mosquito numbers. Also, due to the heterogeneity of the mosquito counts, the data was log transformed to make it amenable to assumptions of the standard normal distribution. The reported differences in attractiveness were then calculated by exponentiating the best fit model parameters for the blend or its variant relative to the humans.
