# Supplementary figures and images for: Development and Field Evaluation of a Synthetic Mosquito Lure That Is More Attractive than Humans
Source: PLoS One. 2010 Jan 28;5(1):e8951. doi: 10.1371/journal.pone.0008951 (PMC2812511; doi:10.1371/journal.pone.0008951)

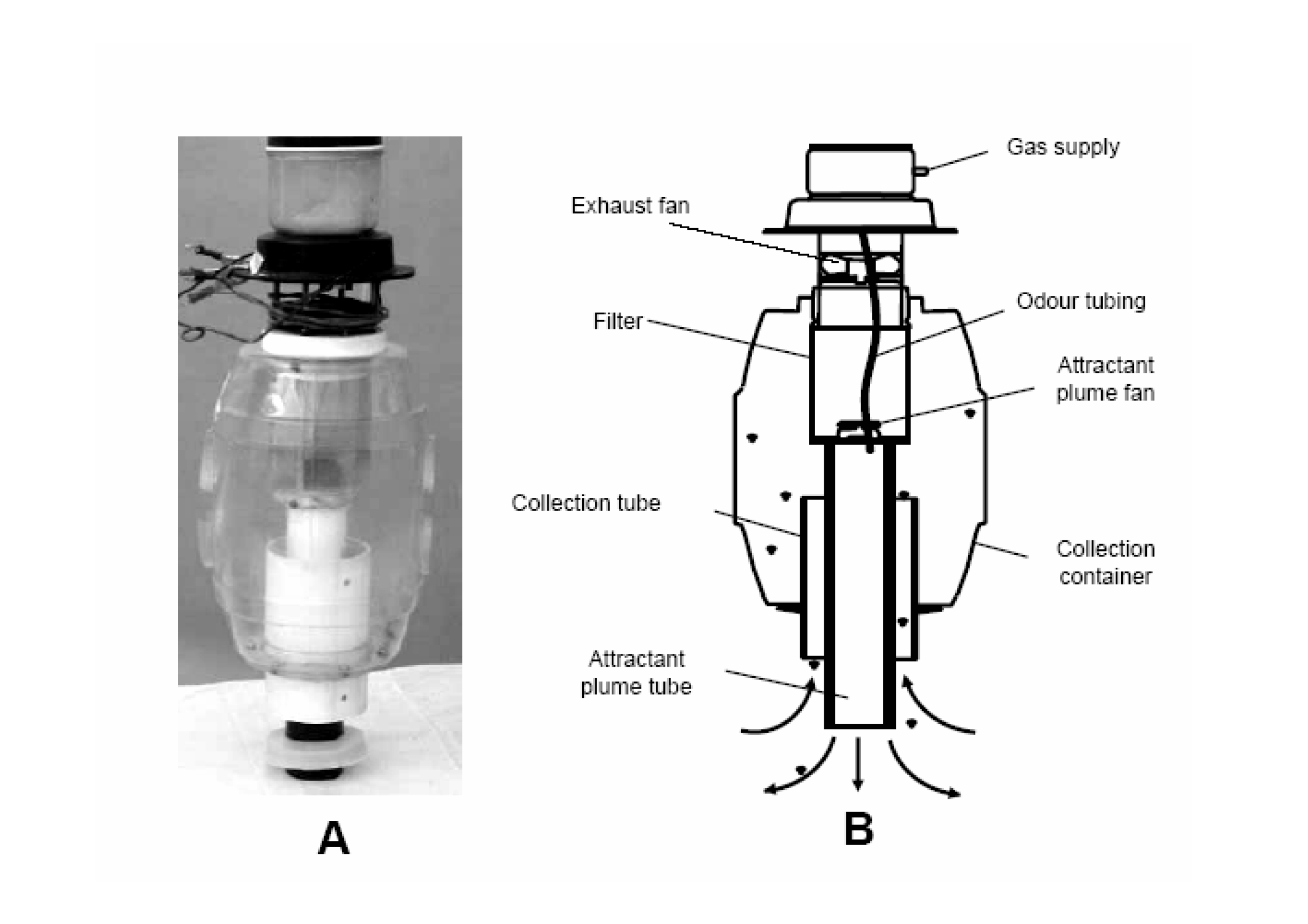

Supplement: Figure S1 — Picture (A) and Drawing (B) of the MM-X® trap. © American Biophysics Corporation. (1.08 MB TIF) [file pone.0008951.s002.tif]

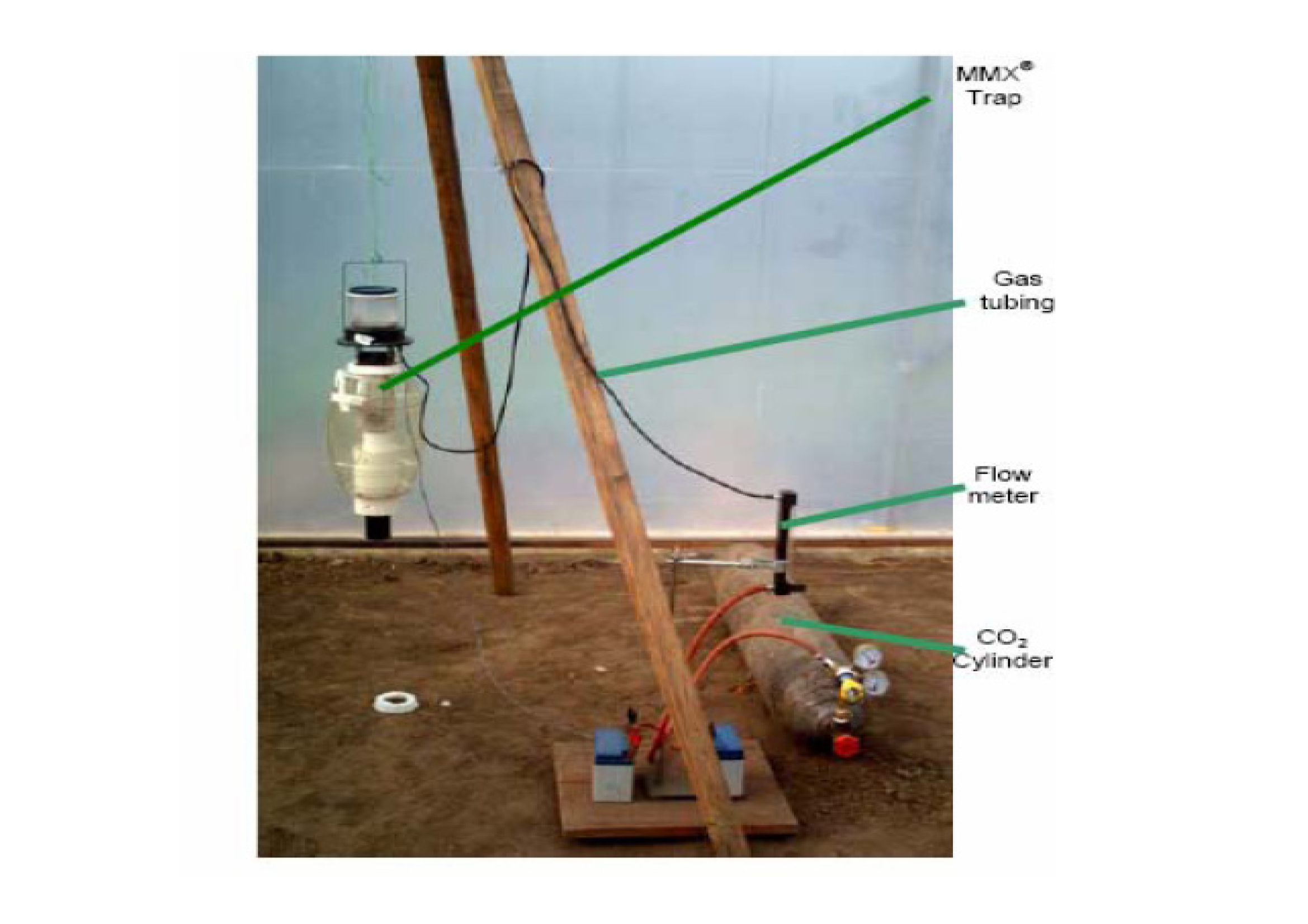

Supplement: Figure S2 — Installation of the MM-X® trap showing how CO2, one of the blend constituents, was delivered to the trap. (4.53 MB TIF) [file pone.0008951.s003.tif]

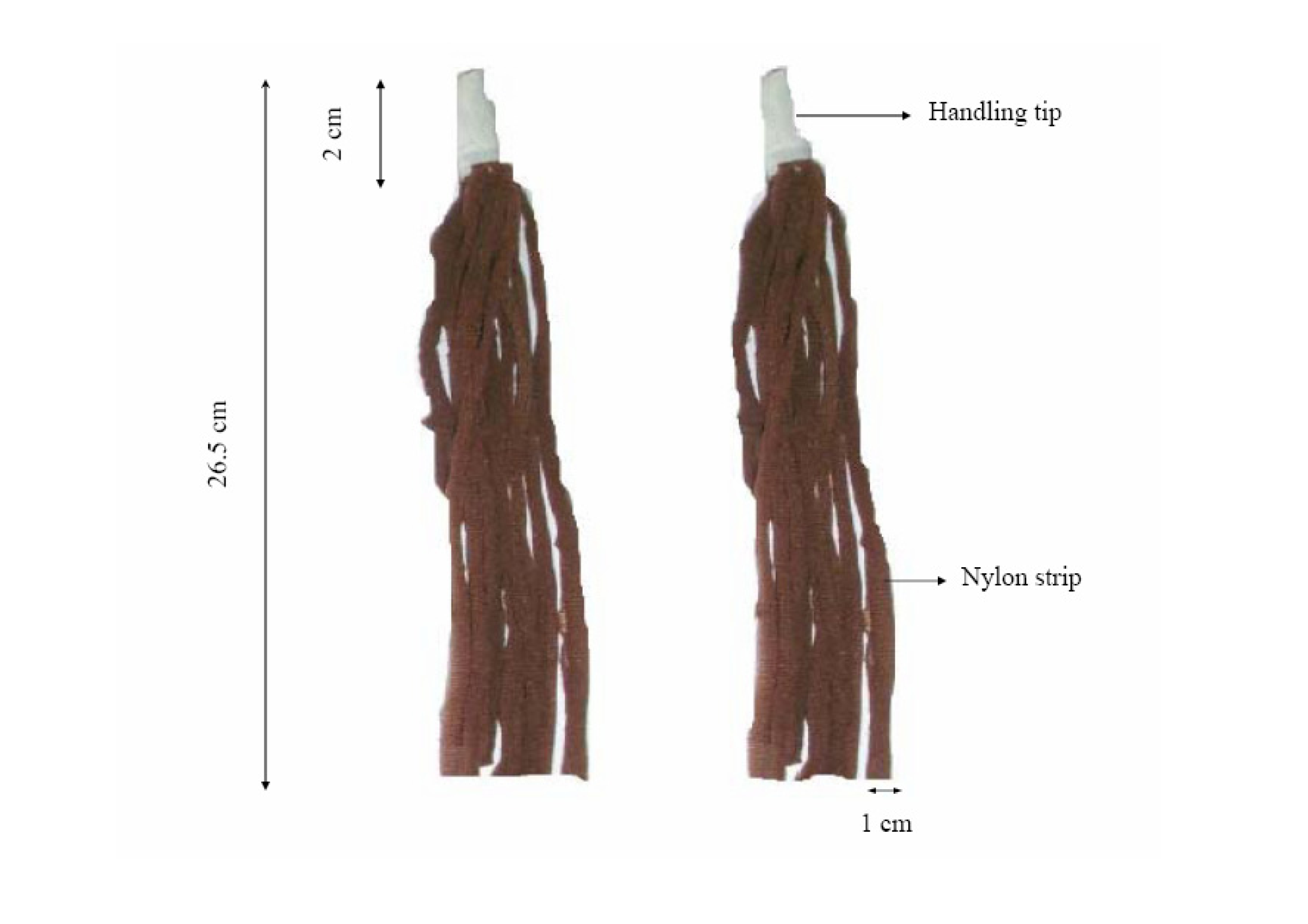

Supplement: Figure S3 — Nylon strips to dispensing the synthetic odor blend. The strips were soaked in the individual test compounds, then removed and kept at 24°C for 4 to 6 hours, so that they were semi-dry by the start of the experiment. To bait the traps, the strips were batched together and inserted into the attractant plume tube of the MM-X® trap. (2.10 MB TIF) [file pone.0008951.s004.tif]

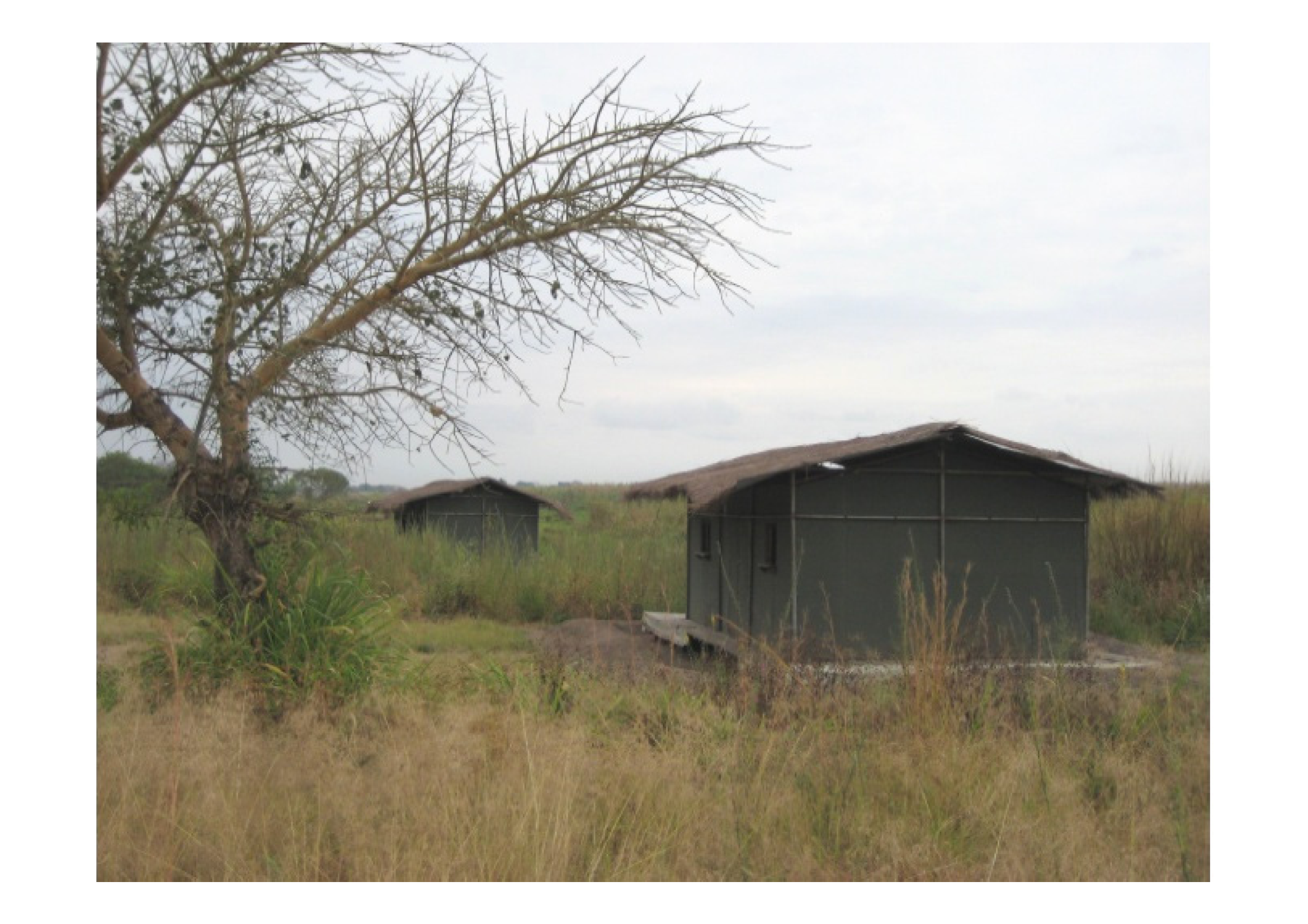

Supplement: Figure S4 — Experimental huts used for field evaluation of the synthetic blend. Four of these huts were used. The experimental huts were similar in average-dimension and shape to the local huts in the study area. They had a galvanized iron frame-work and corrugated iron sheet roofs overlaid with thatch walls were made of canvas on the outside and wood panels coated with mud on the inside. Each hut had one door, two windows and open eaves all round, similar to the local huts, and the daily indoor temperatures in the experimental huts were comparable to that in the local huts, averaging at 28°C. The four huts used for this study were positioned pair wise, such that the distance between two huts of the same pair was 10metres while the distance between the pairs was approximately 100metres. (6.94 MB TIF) [file pone.0008951.s005.tif]
